# Man Transformed: Or the Artificial Changeling

**Published:** 1904-09-10

**Authors:** 


### MAN TRANSFORMED: OR THE ARTIFICIAL CHANGELING.

Burton's "Anatomy of Melancholy" is known at least by reputation to every educated English reader, but it is not generally recognised that his type of mind was not uncommon in the seventeenth century. Burton dwelt rather upon the metaphysical side in his "Anatomy of Melancholy." Bulwer treats of the physical side in his "Anthropometamorphosis," a book which is comparatively little known, but is so entertaining that a short account of it is here given. The second edition of the book is a small quarto, published in London in 1653, entitled "Anthropometamorphosis: Man Transformed: or the Artificial Changeling Historically Presented in the Mad and Cruell Gallantry, Foolish Bravery, Ridiculous Beauty, Filthy Finesse, and Loathsome Loveliness of Most Nations Fashioning and Altering their Bodies from the Mould Intended by nature: with figures of those transfigurations, to which artificiall and affected Deformations are added all the Native and Nationall monstrosities that have appeared to disfigure the humane fabrick. With a Vindication of the Regular Beauty and Honesty of Nature and an Appendix of the Pedigree of the English Gallant." The book starts from the proposition that man was formed in the image of God; "yet the blind impiety of some hath led them to such a height of presumption as to find fault with many parts of this curious fabrick and to question the wisdom of God in the contrivance thereof, upon such blasphemous fancies men have taken upon them an audacious art to forme and new shape themselves, altering the humane figure and moulding it according to their own will and arbitrement, varying it after a wonderful manner, almost every nation having a particular whimsy as touching corporall fashions of their own invention." From this text Dr. Bulwer preaches, collecting his facts from every conceivable source and filling his book with quotations from many authors. He divides his book into scenes and the first scene deals with "Certaine fashions of the head affected and contrived by the pragmaticall invention and artificiall endeavours of many nations." He deals with the methods in use for shaping the heads of children in different countries and by means of woodcuts delineates the different fashions. Like Sir Thomas Brown in his Vulgar Errors he has a sneaking love for all travellers' tales whilst he affects to disbelieve them, and recalls with circumstantial detail the Cynocephali and men whose heads do grow beneath their shoulders. The second scene deals with "certain fashions of the hair affected by divers nations." He then discusses what he calls "frontal fashions" and "eyebrow rites," which is followed by an interesting scene "on Eye-lid Fashions as notes of Gallantry and Beauty by Divers Nations." Dr. Bulwer fitly rounds off this chapter with the reflection that "all endeavour of art pretending to advance the eye above its natural beauty is vain and impious, as much derogating from the wisdom of nature." In like manner he deals with "certain forms and strange shapes of the nose much affected and artificially contrived": "auricular fashions"; "artificial scars accounted marks of gallantry"; "lip gallantry," and so on, through "pap fashions" and "dangerous fashions" under which are placed "desperate affectations about the breast and waist." Then tailed nations are dealt with, and afterwards leg and foot fashions, the book closing with "Cruel and fantastical inventions of men practised upon their bodies in a supposed way of bravery and wicked practices both of men and devils to alter and deform the human fabrick." Added to the work as an appendix is The Pedigree of an English Gallant, wherein the author strives with the fashions of clothes both in men and women, and shows wherein the monstrous fashions then in use were derived from barbarous nations. "To conclude," he says: "touching these indifferent things as cloaths and garments, whosoever will reduce them to their true end must fit them to the service and commodity of the body, whence dependeth their original grace and comliness, which can no way better be done than by cutting them according to the natural shape and proportion of the body, as we may probably imagine the skin garments were, wherewith the Lord God who best knew their shape, first clothed the nakedness of our first parents. What use is there of any than arming sleeves, which answer the proportion of the arme? Or to what end are our breeches as wide at the knee as the whole circumference of the waist? Or why so long as to make men duck-legged. . . . To what end do boots and boot-hose tops appear in that circumference between our legs that we are fain to use a wheeling stride and to go as it were, *in orbe*, to the no little hindrance of progressive motion, which the stradling French basely imitates. . . . It is a wonderful testimony of the imbecility of our judgments, that when we have hit off a convenient fashion we cannot keep to it; but we must commend and allow of fashions for the rareness or novelty, though neither goodness nor profit be joined to them."

